# Derivation of closed-form ellipsoidal X-ray mirror shapes from Fermat’s principle

**DOI:** 10.1107/S1600577522005793

**Published:** 2022-06-20

**Authors:** Kenneth A. Goldberg

**Affiliations:** a Advanced Light Source, Lawrence Berkeley National Laboratory, 1 Cyclotron Road, Berkeley, CA 94720, USA

**Keywords:** X-ray, mirror, ellipsoid, ellipse, Fermat, series, focusing

## Abstract

Ellipsoidal and plane-elliptical surfaces are widely used as reflective, point-to-point focusing elements in many optical systems, including X-ray optics. Here the classical optical path function approach of Fermat is applied to derive a closed-form expression for these surfaces that are uniquely described by the object and image distances and the angle of incidence at a point on a mirror surface. A compact description facilitates design, modeling, fabrication, and testing to arbitrary accuracy. Congruent surfaces in two useful coordinate systems — a system centered on the ellipsoid’s axes of symmetry and a mirror-centered or ‘vertex’ system with the surface tangent to the *xy* plane at the mirror’s center — are presented. Expressions for the local slope and radii of curvature are derived from the result, and the first several terms of the Maclauren series expansion are provided about the mirror center.

## Abstract 2

The derivation of closed-form expressions for ellipsoidal mirror surfaces from the Fermat principle, in terms of the object and image distances, and the glancing angle of incidence, in mirror-centered coordinates is given.

## 1. Introduction

Elliptical and ellipsoidal surfaces are commonly used in reflective optical systems where they ideally focus a point object to a point image. They are conic sections in the plane of incidence, with the object and image points occurring at the foci of an ellipse. They are especially important in X-ray optics where high reflectivities occur at glancing angles of incidence (Compton, 1923 ▸).

**Figure 1:**
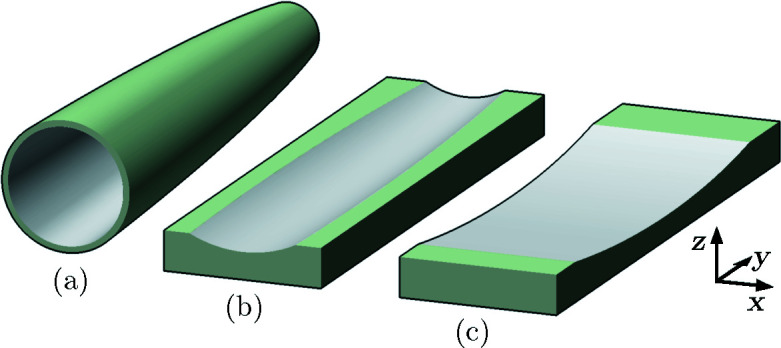
Three types of elliptical mirror elements: (*a*) annular ellipsoid, (*b*) ellipsoidal surface, (*c*) plane-elliptical surface. Shapes (*a*) and (*b*) are portions of a parent ellipsoidal surface. The coordinate axes used in these descriptions are shown.

Several types of elliptical surfaces are now used in optical systems designed for X-rays and other wavelengths (Fig. 1 ▸). Plane-elliptical mirrors, also called elliptical cylinders, have an elliptical profile in the meridional plane (*i.e.* tangentially, along the beam-propagation direction) and are uniform in the sagittal (transverse) direction [Fig. 1 ▸(*c*)]. Such mirrors can be used individually for one-dimensional focusing, and they can be used in orthogonally oriented pairs. The Kirkpatrick–Baez configuration uses two such sequential plane-elliptical mirrors to form an anamorphic, two-dimensional imaging system (Baez & Kirkpatrick, 1948 ▸). The ‘nested’ Montel configuration places orthogonal plane-elliptical mirrors together, side-by-side (Liu *et al.*, 2011 ▸).

With sagittal curvature, a single ellipsoidal mirror can be used for two-dimensional focusing in either an off-axis configuration [Fig. 1 ▸(*b*)] (Yumoto *et al.*, 2017 ▸) or on-axis, with an annular shape [Fig. 1 ▸(*a*)] (Takeo *et al.*, 2020 ▸). Rotational symmetry around the major axis, connecting the foci (*i.e.* the object and image points), requires that the sagittal cross-sections must be circular in planes perpendicular to this axis. The plane-elliptical and ellipsoidal mirror types share the underlying meridional elliptical profile as a surface of rotation.

Ellipsoidal shapes can be described mathematically in a number of ways. Solving for the generating ellipse (in a plane) with fixed object and image distances (or positions) yields a family of ellipses with varying eccentricities, sharing the foci. For each such ellipse, the total ray path length from the object to the image, with a single surface reflection, will be equivalent. Specifying the glancing angle of incidence at one point constrains the solution to a single ellipse with fixed major and minor axes. Importantly, we note that at all points on an ellipsoidal surface the local sagittal and meridional radii of curvature satisfy the Coddington equations for paraxial focusing (Kingslake, 1994 ▸), given the spatially varying distances and incidence angle.

In practice, X-ray mirror surfaces subtend a portion of the parent ellipse or ellipsoid; the point at which they satisfy the glancing angle condition can be taken as the center of the mirror surface, but that is not required.

In the proceeding, we apply the classical optical path function approach that arises from Fermat’s principle (Shaw, 1965 ▸) to write and solve a description of the surface in a mirror-centered coordinate system. In Section 5, the closed-form representation is used to derive a polynomial series approximation. Alternate, published surface descriptions have been based on coordinate transformations, and are discussed in Section 6.

## 2. Two ellipse representations

Ellipses can be described using quadratic polynomials in a two-dimensional plane. In three dimensions, an ellipsoid is a surface of revolution with an ellipse as its generatrix, with the line passing through the foci (containing the major axis) as the axis of rotation.

**Figure 2:**
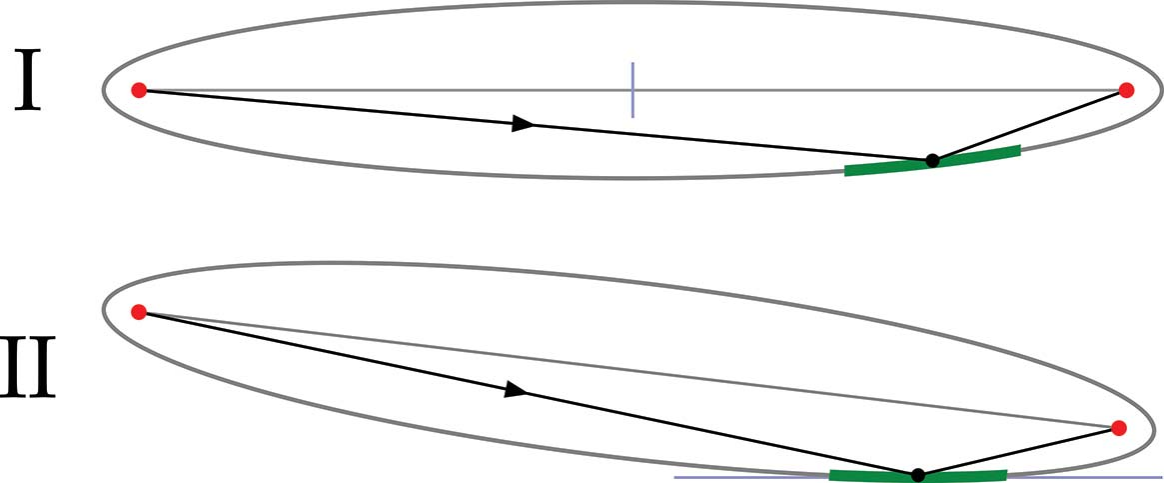
Two classifications of the same parent ellipse: Types I and II. Object and image positions are shown at the foci. The mirror portion, containing the point of intersection, is shown as a thicker segment of the parent ellipse, in green.

We develop two congruent solutions for generalized ellipse descriptions, differing by coordinate transformations, limited to rotation and translation. Shown in Fig. 2 ▸, we refer to these as Types I and II. We use the convention that *x* is the sagittal direction, *y* is the direction of propagation (horizontal on the page), and *z* is the surface height direction (vertical on the page).

The Type I ellipsoid has its major and minor axes aligned with the coordinate *y* and *z* axes, respectively, and is centered at the origin. Type II is tangent to the *xy*-plane at the central point of intersection. In both cases, the sagittal coordinate, *x*, projects outward from the page.

For an X-ray optical system, the most convenient functional description specifies the object distance, image distance, and the central angle of incidence as design parameters. These parameters are here labeled {*p*, *q*, θ}, respectively, with θ defined from the glancing condition. [In other works, these parameters appear as {*r*, *r*′, θ} (McKinney & Palmer, 1997 ▸; Howells *et al.*, 2000 ▸; McKinney *et al.*, 2011 ▸) or as {*R*_2_, *R*_1_, θ} (Yashchuk *et al.*, 2018 ▸, 2019 ▸).]

Following a commonly used coordinate system in X-ray optics, we define the mirror surface height *z* as a function of *x*, the sagittal (transverse) coordinate, and *y*, the longitudinal (tangential) coordinate, with +*y* oriented in the general direction of propagation. The mirror surface is tangent to the *xy* plane at the origin.

Ellipses are described by a conventional set of parameters that includes the major and minor axes, the eccentricity, and the linear eccentricity. Since the ellipses in the two coordinate descriptions are congruent, we can extract these parameters from the Type I description, where they are easier to compute, and apply them to the Type II case.

## 3. Type I ellipsoids

**Figure 3:**
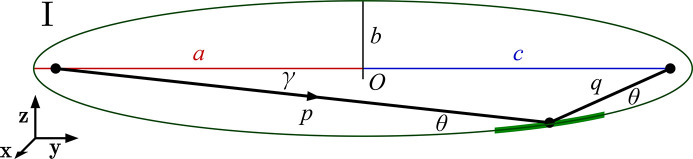
Cross-section of the Type I ellipsoid in the *yz* plane, centered on the coordinate origin, *O*. The direction of light propagation is indicated.

Type I ellipsoids are centered on the coordinate system origin and the axes of the ellipse are aligned with the coordinate axes (Fig. 3 ▸). With circular cross-sections in planes normal to the *y*-axis, the ellipsoidal shape description is well known, and can be represented by 

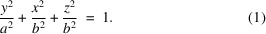

The semi-major axis, *a*, runs along the general beam propagation direction, +*y*. The semi-minor axis is *b* in both transverse directions, *x* and *z*. Every ray leaving the object point must be reflected toward the image point. Rotational symmetry about the major axis therefore requires circular cross-sections: the minor axes in the *x* and *z* directions are equivalent. The elliptical curve defined in the meridional, *yz* plane generates the surface by rotation about the major axis.

For an X-ray optical system design, the object and image distances, *p* and *q*, respectively, are commonly dependent on the magnification requirements, *M* = *q*/*p*, while the glancing angle of incidence θ is set by reflectivity considerations. The values of *a* and *b* are not given in advance.

We can write the surface solution explicitly in this coordinate system, solving for *z* from equation (1). To have an upward-facing (+*z*) mirror, we choose the negative root of equation (2). 

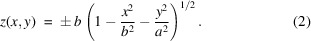

Howells *et al.* (2000 ▸) provide geometric relationships among the ellipse parameters for any cross-section containing the *y*-axis. First, the major axis *a* is 

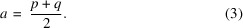

Consulting Fig. 3 ▸, the linear or arithmetic eccentricity, *c*, can be derived from the Law of Cosines [as shown by McKinney *et al.* (2011 ▸)], 

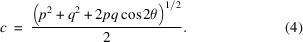

The two foci are at (0, −*c*, 0) and (0, *c*, 0). The square of the semi-minor axis is given by *b* ^2^ = *a* ^2^ − *c* ^2^. With equations (3) and (4), this reduces to 

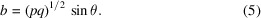

The Type I surface in equation (2) can now be written as a function with parameters *p*, *q*, and θ,

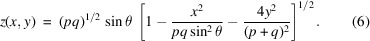

The ellipse’s eccentricity, *e*, is 

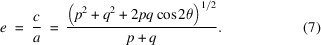

By congruence, we note that the relations that define *a*, *b*, *c*, and *e* in terms of *p*, *q*, and θ hold for all translations and rotations of a Type I ellipsoid, including the Type II ellipsoid described in the following section.

In the *yz* plane, the point of intersection on the surface (0, *y*_0_, *z*_0_) can be derived from the Law of Sines and the relations above. The incident ray declines at an angle γ from the *y*-axis (Fig. 3 ▸),

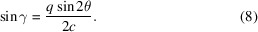

Recognizing that 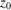 = 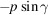 , we substitute equations (4) and (8),

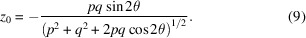

The *y*_0_ value is solved from equation (1), with *a* and *b* from equations (3) and (5). After reduction, 

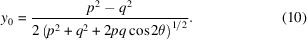

In a more compact form, 

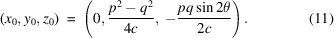

Note that when *p* = *q* (unity magnification), symmetry demands that *y*_0_ = 0, and the eccentricity reduces to *e* = 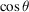 .

The slope of the mirror at the central point of intersection, μ, is 

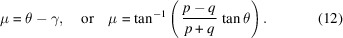

The equivalent second expression was derived by Yashchuk *et al.* (2019 ▸) [equation (11) therein].

## 4. Type II ellipsoids

The Type II ellipsoid geometry is the most convenient description for X-ray optics design, modeling, manufacturing and testing. With the central point of intersection at the origin, and the surface tangent to the *xy* plane at that location, the mirror shape has a minimal net slope across its length.

**Figure 4:**
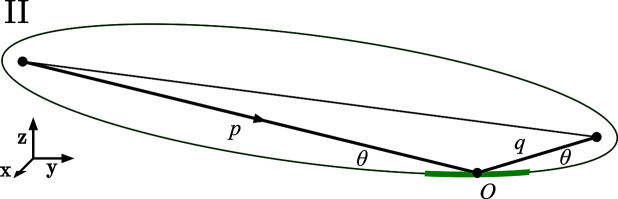
The Type II ellipsoid geometry is the most convenient description for modeling, fabrication, and testing. The coordinate origin is placed at the central point of intersection, where the surface is tangent to the *xy* plane. Shown is the cross section through the *yz*, meridional plane. The direction of light propagation is indicated.

Taking a cross-section of the surface in the *yz* plane, the coordinate system and geometry are shown in Fig. 4 ▸. The two foci of the ellipse are the object point, 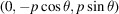 , and the image point, 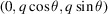 .

Here we solve the ellipsoid with the optical path function approach, applying the constant path-length constraint that arises from the Fermat principle. This approach was described for other optical systems by McKinney & Palmer (1997 ▸). The result is a solution valid to arbitrary precision.

The ellipsoidal and the plane-elliptical surfaces can be generated from the same representation, differing only by the treatment of the *x* coordinate. For plane-ellipses, removing the *x* dependence makes the surface uniform in *x*.

The solution proceeds as follows. For a point (*x*, *y*, *z*) on the surface of the ellipsoid, a constant total path length requires that the sum of the distances from the foci to the surface be constant and therefore equal to the distance at the central point, 

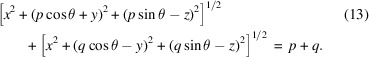

Solving *z*(*x*, *y*) requires isolating square roots and squaring the equation two times. As the expressions are expanded, many terms cancel. Gathering the powers of *z*, which occur up to second order, allows solution with the quadratic formula. For 

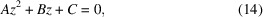

the solutions are 

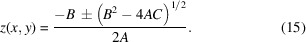

Expansion of (13) leads to equation (16). We define a recurring term, *h* = 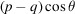 ,

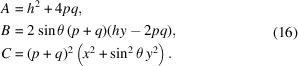

Following reduction, the complete solution of the Type II ellipse is 

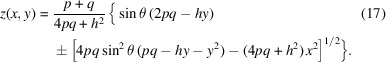

We choose the negative root for the concave, upward-facing portion of the surface.

From equation (17), the tangential shape along the *x* = 0, *yz* plane is the shape of the related plane-ellipse,

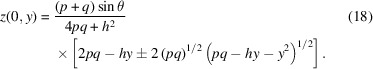

The sagittal shape along the *y* = 0, *xz* plane is also an ellipse,

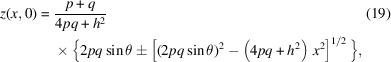




### 4.1. Slope and radius of curvature

The slope and curvature, and their variation across the surface, are important to the fabrication and testing of X-ray mirrors. The meridional slope along the center-line, in the *yz* plane (often referred to as the *tangential* direction), can be computed from the first derivative of equation (18) with respect to *y*,

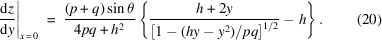

The curvature is closely related to the second derivative, being its inverse when the slope is zero (*e.g.* at the origin),

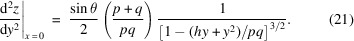

We can use equation (17) to compute the variation in the sagittal radius of curvature along the length of a mirror,

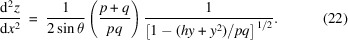

From equations (21) and (22), we recognize that the radii of curvature match the familiar Coddington radii (Kingslake, 1994 ▸) at the origin. For the meridional and sagittal radii, 

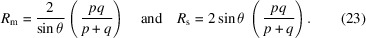

The *pq*/(*p* + *q*) term is the paraxial focal length of the mirror, defined below. Furthermore, since the mirror’s center-point is arbitrary on the parent ellipsoid, we know that, locally, *R*_m_(*y*) will match this form as *p*, *q*, and θ vary along the mirror surface.

## 5. Series expansion

Many authors have used polynomial series expansions about the mirror center to describe elliptical and ellipsoidal surface shapes (Howells, 1980 ▸; Rah & Howells, 1997 ▸; Rah *et al.*, 1997 ▸; Peatman, 1997 ▸; McKinney *et al.*, 2011 ▸; Yashchuk *et al.*, 2019 ▸). This mathematical approach simplifies understanding of central curvatures and can provide an approximation to surface shapes when closed-form representations are not available. Series expansions have also been used to facilitate solutions for mechanically bent mirror substrates, connecting the shape description to beam-bending equations (Rah *et al.*, 1997 ▸; Howells *et al.*, 2000 ▸; Zhang *et al.*, 2010 ▸; Yashchuk *et al.*, 2018 ▸).

A conventional Maclauren series expansion in orders of *x* and *y* takes the form 

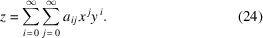

The series expansion of equation (17) was calculated with *Mathematica* (Wolfram Research, 2020 ▸), simplified, and tested empirically. The coefficients up to fourth order, (*i* + *j*) ≤ 4, are listed in equations (26). We place the coordinate origin at the center of the mirror, with zero height (*a*_00_ = 0). With the surface tangent to the *xy* plane at that point, the first-order (slope) terms (*a*_10_ and *a*_01_) are also zero. Symmetry about the meridional (*yz*) plane dictates that odd-ordered terms in *x* must also be zero.

It is helpful to define the paraxial focal-length, *f*, which appears as a factor in each coefficient, 

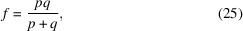




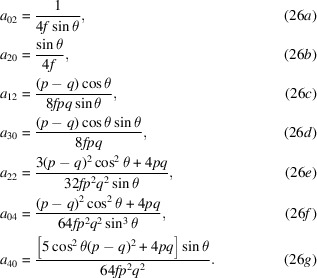

For approximating plane-elliptical surfaces with no sagittal curvature, set *a*_*ij*_ coefficients with *j* > 0 to zero.

Analysis in cases relevant to soft X-ray geometries shows that fourth-order series approximations may be accurate to tenths of a nanometer with the largest discrepancies in the corners of the domain, where beam intensities may be low.

## 6. Alternative solutions

Several previous authors have offered solutions to the ellipsoidal or plane-elliptical shape relevant for X-ray optical designs, and these expressions take a variety of forms. The accuracy of any solution is numerically verifiable by calculating the total path length from the object to the image point, with a single reflection, considering all points on the surface: the distance should be uniformly equal to *p* + *q*. Indeed, while other solutions are derived from coordinate transformations, equation (17) flows directly from the constant path length requirement of Fermat’s principle.

For X-ray optics, the earliest published expression for the ellipsoidal surface, and the resulting slopes and curvatures, may have been given by Rah *et al.* (1997 ▸), using a set of expressions based on the conjugate distances and the central angle. Although the form of that equation is significantly different than equation (17), it produces identical results numerically. It is not clear from the context or the references how it was derived.

Rommeveaux presented a geometric (2D) ellipse solution (Rommeveaux *et al.*, 2007 ▸) based on coordinate rotation and translation from the Type I ellipse to the mirror-centered coordinates. The solution utilizes the central slope of the mirror (in the Type I coordinate system), μ, that is not solved in the text, but is given here in equation (12).

In the context of optimized mirror bending, McKinney *et al.* (2011 ▸) also derived a planar ellipse description using a coordinate transformation and a quadratic expression for the elliptical shape. The mirror height function, equation (B6) therein, contains an error of an omitted *x*. The final term in the numerator should be 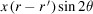 , and when corrected produces numerical results identical to equation (17). McKinney *et al.* also comment on the relation of the solution to the paraboloid case where *p* → ∞.

More recently, Yashchuk *et al.* (2019 ▸) provided a planar ellipse solution involving a nested coordinate transformation, from the Type I frame. In the *x* = 0 plane, it also proves to be numerically identical to equation (17).

The reader is advised that some authors define the incident angle from the surface normal, not by the glancing angle as defined here.

## 7. Summary

We have derived closed-form expressions for ellipsoidal surfaces most applicable in reflective optics, and X-ray optics in particular, where these surfaces are widely used. Arising from Fermat’s principle, the equations are based on the object distance, image distance, and glancing angle of incidence at the center of the mirror, {*p*, *q*, θ}. Representation in the mirror-centered coordinate system is most convenient for design, modeling, fabrication, and testing; while a representation of the congruent ellipsoid in the coordinate system where it is centered and aligned with the axes simplifies the extraction of the widely used ellipse parameters, *a*, *b*, *c*, and *e*.

Polynomial series approximations of the surface shape are useful in many applications, and can simplify the extraction of curvatures and slopes, yet the closed-form expression enables calculations to be made with arbitrarily high accuracy for computation.

Path-length optimization is commonly used to design or study complex optical systems with one or more optical surfaces. Yet tracing arbitrary rays through multiple reflections (or refractions) poses a challenge for analytical methods, except in paraxial cases or with special surfaces. The analytical approach applied here is effective largely because the input and output waves are simple and there is only one reflection to consider.
